# Cow-baited tents are highly effective in sampling diverse *Anopheles* malaria vectors in Cambodia

**DOI:** 10.1186/s12936-016-1488-y

**Published:** 2016-08-30

**Authors:** Brandyce St. Laurent, Kolthida Oy, Becky Miller, Elizabeth B. Gasteiger, Eunjae Lee, Siv Sovannaroth, Robert W. Gwadz, Jennifer M. Anderson, Rick M. Fairhurst

**Affiliations:** 1 Laboratory of Malaria and Vector Research, National Institute of Allergy and Infectious Diseases, National Institutes of Health, Rockville, MD 20852 USA; 2 National Center for Parasitology, Entomology, and Malaria Control, Phnom Penh, 12101 Cambodia

**Keywords:** *Anopheles*, Cambodia, Trap, Outdoor transmission, Malaria, Vector

## Abstract

**Background:**

The accurate monitoring and evaluation of malaria vectors requires efficient sampling. The objective of this study was to compare methods for sampling outdoor-biting *Anopheles* mosquitoes in Cambodia.

**Methods:**

In the Cambodian provinces of Pursat, Preah Vihear, and Ratanakiri, six different mosquito trapping methods were evaluated: human landing collection (HLC), human-baited tent (HBT), cow-baited tent (CBT), CDC miniature light trap (LT), CDC miniature light trap baited with molasses and yeast (LT-M), and barrier fence (F) in a Latin square design during four or six consecutive nights at the height of the malaria transmission season.

**Results:**

Using all traps, a total of 507, 1175, and 615 anophelines were collected in Pursat, Preah Vihear, and Ratanakiri, respectively. CBTs captured 10- to 20-fold more anophelines per night than the other five sampling methods. All 2297 *Anopheles* mosquitoes were morphologically identified and molecularly typed using standard morphological keys and sequencing the rDNA ITS2 region to distinguish cryptic species, respectively. Overall, an extremely diverse set of 27 known *Anopheles* species was sampled. CBTs captured the same molecular species that HLCs and the other four traps did, as well as additional species. Nine specimens representing five *Anopheles* species (*Anopheles hyrcanus*, *Anopheles barbirostris* sensu stricto, *Anopheles barbirostris* clade III, *Anopheles nivipes*, and *Anopheles peditaeniatus*) were infected with *Plasmodium falciparum* and were exclusively captured in CBTs.

**Conclusions:**

These data indicate that cow-baited tents are highly effective in sampling diverse *Anopheles* malaria vectors in Cambodia. This sampling method captured high numbers of anophelines with limited sampling effort and greatly reduced human exposure to mosquito bites compared to the gold-standard human landing collection.

**Electronic supplementary material:**

The online version of this article (doi:10.1186/s12936-016-1488-y) contains supplementary material, which is available to authorized users.

## Background

Malaria transmission in the Greater Mekong Subregion (GMS), where artemisinin-resistant *Plasmodium falciparum* parasites have emerged, endangers global malaria control efforts. As treatment failure rates for frontline anti-malarial drugs continue to worsen [1, 2], control efforts focusing on malaria vectors in the GMS have become increasingly important. Western Cambodia in particular has been a hotspot for the evolution and spread of drug-resistant *P. falciparum* parasites. Since these parasites can infect extremely diverse *Anopheles* species, including the major vector of sub-Saharan Africa, *Anopheles coluzzii* (formerly *Anopheles gambiae* M form) [3], local control and elimination efforts are needed to prevent the spread of these dangerous pathogens to other regions. The natural transmission of these parasites and the efficacy of integrated malaria control efforts in Southeast Asia cannot be characterized without the appropriate sampling of local vectors.

The anopheline vectors in the GMS are incredibly diverse and primarily bite outdoors [4–6], enabling them to avoid commonly used vector control interventions, such as indoor residual sprays or insecticide-treated bed nets. While many different traps have been extensively evaluated in regions of highly-endemic malaria transmission in sub-Saharan Africa, little is known about trapping efficacy in many parts of Southeast Asia.

The human-landing collection (HLC) is considered the best sampling method to estimate human exposure to potentially infectious bites by malaria vectors. However, in many regions where vector density is low or vector species demonstrate generalist host-feeding behaviours, the yields of sampling methods like HLCs are insufficient to adequately sample the vector population. In addition, HLCs are extremely labour-intensive and may expose collectors to potentially infectious bites. Several alternatives to HLC have been explored in regions in sub-Saharan Africa and Asia, typically involving a protected human contained in a larger net or trap [7–9]. Other attractive traps include the Centers for Disease Control and Prevention (CDC) miniature light trap, which attracts mosquitoes with a light and sometimes CO_2_ bait, which in some cases can yield results comparable to those of the HLC [10]. While not labour-intensive, light traps require a battery and a CO_2_ or odor source to attract mosquitoes. Passive trapping techniques for outdoor-biting malaria vectors, such as a barrier fence, are currently being explored as monitoring and control tools [11]. Low-cost collection techniques that effectively sample malaria vectors are critical for evaluating transmission.

Although a number of thorough surveys have been conducted in the GMS [4, 12, 13], many studies have not screened “secondary” vectors for *Plasmodium* infection, but rather screened only the presumed “primary” vectors: *Anopheles dirus*, *Anopheles minimus*, and *Anopheles maculatus*.

Since sampling efficacy and mosquito species distributions can differ by location, trap efficiency was evaluated in three different Cambodian provinces where clusters of clinical malaria cases had been identified during previous peak transmission seasons.

## Methods

### Study sites

From July to August 2013, mosquitoes were collected in a single village in each of three Cambodian provinces. Sayas Village, Ratanakiri (13°32′51.5″N 107°01′28.4″E) is an isolated community in the forest. Chean Mok commune, Preah Vihear (13°46′11.3″N 104°55′13.4″E) is a small community at the base of a mountain. Ankrong Village, Pursat (12°18′46.5″N 103°34′13.4″E) is a rural farming community approximately 40 km outside of Pursat town. Villages were selected based on clustering of clinical *P. falciparum* malaria cases during previous transmission seasons.

### Mosquito sampling

Adult mosquitoes were collected using six different outdoor sampling methods concurrently at each site:

- Cow-baited tent (CBT) collections took place from 6 p.m. to 6 a.m. An adult cow was loosely tied to a stake in the ground at the centre of a large tent (Coleman 13 × 15 ft. screened canopy tent). Mosquitoes resting on the interior walls of the tent were collected hourly for 5 min using mouth aspirators. Human-baited tent (HBT) collections were done similarly, but the human was protected from mosquito bites using a smaller tent (Eureka! solitaire 1-person tent) inside of the same larger tent.
- Human landing collections (HLC) took place from 6 p.m. to 6 a.m. Trained collectors sat with their legs exposed and collected mosquitoes landing on their skin using a mouth aspirator and a flashlight. Human landing collectors sat outside in a dark area and stored mosquitoes in a paper cup, which was collected hourly. Collectors worked in 6-h shifts, switching out with other collectors at midnight, in accordance with the Cambodian National Center for Parasitology, Entomology, and Malaria Control HLC protocol.
- CDC miniature light traps (LT) (John Hock Co., Gainesville, FL) and the same light traps baited with a mixture of molasses and yeast (LT-M) as a source of CO_2_ [14] were placed outdoors at sampling locations. The LT contains a battery-powered light and fan. Mosquitoes near the fan are sucked into the trap. In this case, the molasses and yeast release CO_2_ as a lure and the unbaited trap acts as a control with only the light serving as the attractant. Mosquitoes were collected from the LTs every morning between 5:30 a.m. and 6 a.m.
- A barrier-screened fence that was not treated with insecticide was erected between the village and the forest. The barrier fences were 20 m long and 2 m high. Two collectors simultaneously sampled the inner, village-facing side of the fence (F-I), and the outer, forest-facing side of the fence (F-O) in a single pass every hour from 6 p.m. to 6 a.m. In Ratanakiri, collections from both sides of the fence were pooled (F).

Three locally representative sites per village were selected for the experiment and sets of traps were rotated through these locations at least 30 m apart over consecutive nights. Trap locations at least 15 m from other host attractive cues were selected. An LT and LT-M remained at all three trapping locations each night of collection. HLCs, CBTs, and HBTs were rotated through the three locations every night at least 10 m from either LT. Individual mosquitoes were held separately in paper cups according to collection method and hour, and then morphologically identified in the field. Mosquitoes were stored individually in barcoded 1.5-ml tubes with silica gel desiccant until they were molecularly analysed.

### Mosquito identification and molecular analyses

Genomic DNA was isolated from individual mosquito heads and thoraces using a CTAB-based DNA extraction method. The ribosomal DNA internal transcribed spacer region (rDNA ITS2) was PCR-amplified using ITS2A and ITS2B primers [15] that were developed to differentiate *Anopheles* cryptic species, and then sequenced. PCR products were visualized on a 1 % agarose gel and purified by mixing 8 μl of PCR product with 2U of exonuclease 1 (USB Corporation, Cleveland, OH), 1U of shrimp alkaline phosphatase (USB Corporation), and 1.8 μl of ddH_2_0. This cleanup mixture was incubated at 37 °C for 15 min, and then at 80 °C for 15 min to inactivate the enzymes. PCR products were sequenced directly using Sanger sequencing on an ABI 3730 xl DNA analyzer platform (PE Applied Biosystems, Warrington, England). Clean ITS2 sequences for each specimen were blasted using BLASTn against the NCBI GenBank nr database to confirm molecular species identification when compared to voucher and published sequences.

Extracted DNA from each individual mosquito head and thorax was used to test for *Plasmodium* infection using a nested PCR to amplify a portion of the *Plasmodium* mitochondrial cytochrome B gene (*cytb*) [16]. The Sanger sequences of these PCR-positive amplicons were assigned to *Plasmodium* species by comparing them to known *Plasmodium* voucher *cytb* sequences in the NCBI database.

Abdomens of mosquitoes that were blood-fed were separated from the head and thorax, and analysed using a multiplex, blood meal-diagnostic PCR assay based on vertebrate mitochondrial *cytb* DNA sequences [17]. Blood meal DNA samples that did not amplify in the diagnostic PCR assay were sequenced and blasted against the NCBI GenBank nr database to identify the source of the blood meal.

### Data analysis

Data from each mosquito collection were analysed using GraphPad Prism software version 6 (GraphPad, San Diego, CA). The mean catch differences between sampling methods at each site were analysed using ANOVA. The catches were treated as the dependent variable and compared using ANOVA with a significance threshold of p ≤ 0.05. Trap yields were compared only within the same village. The null hypothesis was that there was no difference in nightly anopheline catch between sampling methods (no trap effect). A post-hoc Tukey’s HSD test was performed to identify statistically significant differences between total catch due to trap and location effects in the experiment. This analysis was performed on the total number of anophelines captured per night, which was greater than the total number of molecularly analysed anophelines.

## Results

Six different trapping techniques were compared to determine which methods would be appropriate for monitoring outdoor-biting malaria vectors in Cambodia during the peak transmission season. The trapping techniques analysed included cow-baited tents (CBT), human-baited tents (HBT), human landing collections (HLC), CDC miniature light traps (LT), CDC miniature light traps baited with molasses and yeast (LT-M), and a barrier fence (F) with collections on the village-facing (F-I) and forest-facing (F-O) sides of the fence.

Overall, 27 known *Anopheles* species were collected in three provinces (Table 1). These species represent ten diverse species groups, including four species in the *Anopheles annularis* group, five species in the *Anopheles barbirostris* group, four species in the *Anopheles funestus* group, six species in the *Anopheles hyrcanus* group, three species in the *An. maculatus* group, as well as *An. dirus A*, *Anopheles kochi*, *Anopheles splendidus*, *Anopheles tessellatus*, and *Anopheles vagus.* These findings indicate an extreme level of *Anopheles* diversity, even when compared to other areas in the GMS. Many of the molecularly identified species collected were only recently recognized as distinct subspecies, as anopheline species complexes in this region are being more accurately described [18], including several members of the *An. barbirostris* [19], *An. annularis* [20], and *An. hyrcanus* groups [21, 22].

**Table 1:** Molecular identification of 2297 *Anopheles* species according to Cambodian province

Molecular identification	Pursat	Preah Vihear	Ratanakiri	Total
*Annularis* group				
*An. annularis*		1	7	8
*An. nivipes*	106	276	219	601
*An. pallidus*	1	1		2
*An. philippinensis*	47	51	80	178
*Barbirostris* group				
*An. barbirostris*	1	3	4	8
*An. barbirostris (barbirostris* clade III*)*	17	77	51	145
*An. campestris*		1		1
*An. saeungae (barbirostris* clade IV*)*	7	71	79	157
*An. wejchoochotei*		12	1	13
*An. dirus* complex				
*An. dirus A*	2	23	4	29
*Funestus* group				
*An. aconitus*		13	4	17
*An. minimus A*		5	2	7
*An. jeyporiensis*	1		3	4
*An. pampanai*	3		6	9
*Hyrcanus* group				
*An. argyropus*			7	7
*An. crawfordi*	85		1	86
*An. hyrcanus*	1		1	2
*An. peditaeniatus*	4	7	21	32
*An. nigerrimus*			20	20
*An. nitidus*	2		1	3
*Kochi* group				
*An. kochi*	72	130	6	208
*Maculatus* group				
*An. sawadwongporni*	20	19	4	43
*An. rampae*	2	29	18	49
*An. karwari*	13	12	7	32
Other				
*An. splendidus*		8	7	15
*An. tessellatus*	8	32	13	53
*An. vagus*	111	404	49	564
Unknown *Anopheles*	4			4
Total	507	1175	615	2297

The numbers of *Anopheles s*pecies, identified by rDNA ITS2 sequences, collected during a short period in Pursat, Preah Vihear, and Ratanakiri, Cambodia are shown. Members of common cryptic species complexes are arranged by their corresponding species group

**Fig. 1:**
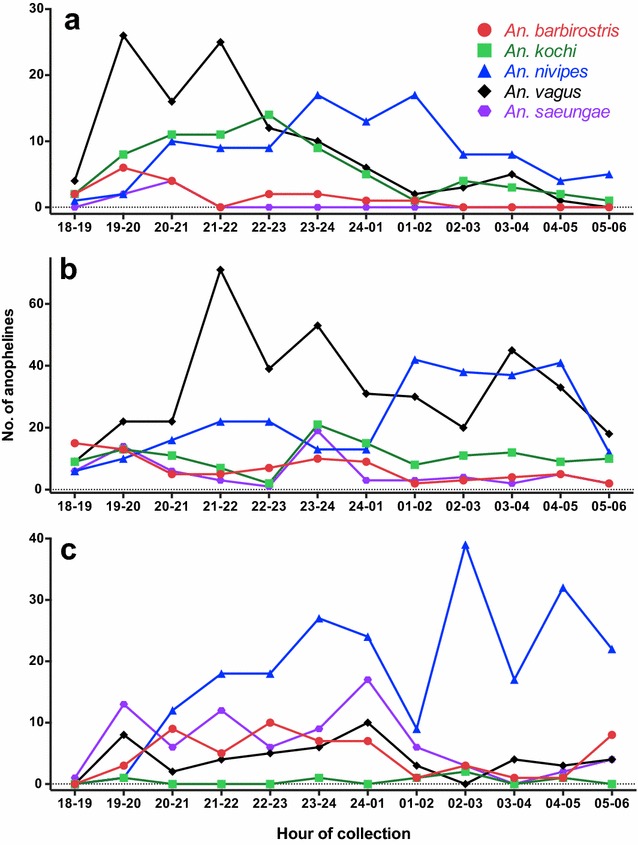
Comparison of total hourly collection of the five most abundant *Anopheles* species sampled in three Cambodian provinces. **a** Pursat, **b** Preah Vihear, and **c** Ratanakiri. The vast majority (90 %) of anophelines were captured in cow-baited tents

The five most prevalent species across the three sites—*Anopheles nivipes* (n = 601), *An. vagus* (n = 564), *An. kochi* (n = 208), and two members of the *An. barbirostris* complex, *An. barbirostris* (n = 145) and *Anopheles saeungae* (n = 157)—represented 73 % of the total collection (Table 1). These were the most common species collected by most of the trapping methods, indicating that they were likely the most abundant species in each sampling site during the sampling period. *Anopheles nivipes*, *An. vagus*, and *An. saeungae* were collected in all trap types (Table 2), with 88, 91 and 94 % of them collected in the CBT, respectively. *Anopheles kochi* were collected in the CBT, HBT, HLC, F-I, and LT-M. *Anopheles barbirostris* (clade III) were predominantly collected in the CBT (140/145, 97 %), while only a few specimens were sampled in the HBT (n = 1) and HLC (n = 4). These five most-prevalent species displayed different biting patterns in the three collection sites. *Anopheles vagus* had peak biting activity early in the night in Pursat and Preah Vihear, but was less prevalent and had no apparent peak biting period in Ratanakiri (Fig. 1). *Anopheles nivipes* appeared to be actively biting later in the night, and in gradually increasing numbers after 10 p.m. *Anopheles saeungae* was abundant in the first half of the night in Preah Vihear and Ratanakiri, but was not prevalent in Pursat. *Anopheles kochi* and *Anopheles barbirostris* seemed to bite throughout the night, though further sampling is necessary to more accurately evaluate their biting behaviours.

**Table 2:** Total species collected according to trap and Cambodian province

Molecular identification	Pursat							Preah Vihear							Ratanakiri					
	CBT	HBT	HLC	F-I	F-O	LT	LT-M	CBT	HBT	HLC	F-I	F-O	LT	LT-M	CBT	HBT	HLC	F	LT	LT-M
*Annularis* group																				
*An. annularis*									1						7					
*An. nivipes*	93	6	3	3		1		247	20	4		1	1	3	191	13	10	5		
*An. pallidus*	1							1												
*An. philippinensis*	44	1	1		1			51							76	3		1		
*Barbirostris* group																				
*An. barbirostris*	1							3							4					
*An. barbirostris (barbirostris* clade III*)*	16		1					75		2					49	1	1			
*An. campestris*								1												
*An. saeungae (barbirostris* clade IV*)*	4		1		1	1		67		1			2	1	76	1		2		
*An. wejchoochotei*								10	2						1					
*An. dirus* complex																				
*An. dirus A*	2							10	7	5		1				3	1			
*Funestus* group																				
*An. aconitus*								11		1				1	4					
*An. minimus A*								2	2	1					1		1			
*An. jeyporiensis*	1														2		1			
*An. pampanai*	3														3		3			
*Hyrcanus* group																				
*An. argyropus*															7					
*An. crawfordi*	77	4	3				1									1				
*An. hyrcanus*	1														1					
*An. peditaeniatus*	4							7							18	1	2			
*An. nigerrimus*															19	1				
*An. nitidus*	2														1					
*Kochi* group																				
*An. kochi*	59	2	8	2			1	128						2	6					
*Maculatus* group																				
*An. sawadwongporni*	14	4	2					14		1			1		4					
*An. rampae*	2							23	1	1			2		17		1			
*An. karwari*	12	0	1												6	1				
*An. splendidus*								8							6	1				
*An. tessellatus*	7	1						27	2	1	2				13					
*An. vagus*	85	10	13	1	1	1		386	3	2	2		6	5	42	6		1		
Unknown *Anopheles*	4																			
Total collected:	432	28	33	6	3	3	2	1071	38	19	4	2	12	12	554	32	20	9	0	0
% of total collected	85	6	7	1	1	1	0	92	3	2	0	0	1	1	90	5	3	1	0	0
No. of species collected:	19	8	9	3	3	3	2	18	8	10	2	2	5	5	23	11	8	4	0	0
Sporozoite positive?	Yes														Yes					

*Anopheles* species collected in Pursat, Preah Vihear, and Ratanakiri provinces according to trap: CBT (cow-baited tent), HBT (human-baited tent), HLC (human landing collection), F-I (fence facing village), F-O (fence facing away from village), F (both sides of fence combined—Ratanakiri only), LT (CDC light trap), LT-M (CDC light trap baited with molasses and yeast). The total number of specimens, % of total collection, number of species collected, and number of *Plasmodium*-positive specimens are shown at the bottom

*Anopheles dirus*, considered the major vector species in the GMS [6, 23], represented only 1 % of the total collection, and were collected in the CBT (n = 12, two sites), HBT (n = 10, two sites), HLC (n = 6, two sites), and F-O (n = 1, one site) (Table 2). *Culex*, *Aedes*, *Toxorhynchites*, and *Mansonia* were also present in these collections, particularly in LT collections.

The method of sampling, but neither the night of collection nor the location of traps, significantly affected the number of anophelines captured in all three provinces. The CBT yielded the highest total catch and *Anopheles* species richness in each province. A total of 2297 anopheline mosquitoes were collected using all trap methods in all three provinces. The CBT collected 90 % (n = 2055) of the total anophelines, while the HBT, HLC, F-I, F-O, LT, and LT-M collected only 4 % (n = 98), 3 % (n = 72), 1 % (n = 19), <1 % (n = 5), 1 % (n = 15), and 1 % (n = 14) of the total anophelines, respectively.

**Fig. 2:**
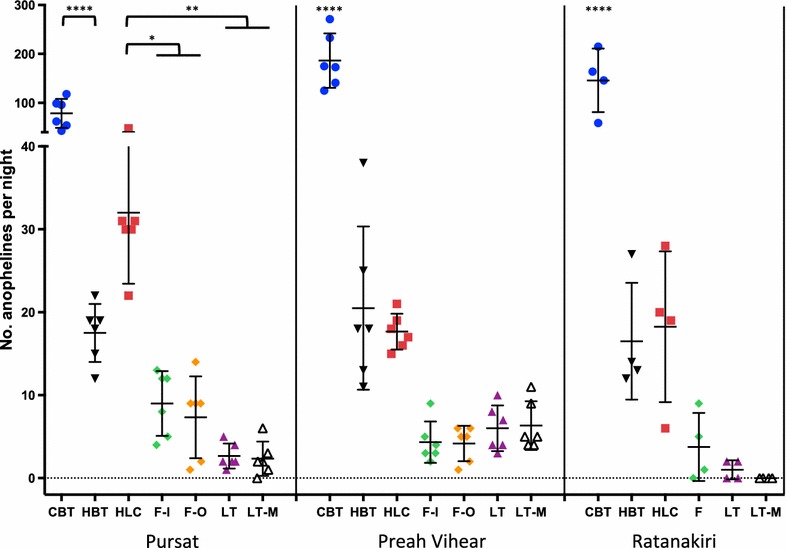
Number of anophelines captured per night using six collection methods in three Cambodian provinces. Each *dot* represents the total number of anophelines collected in a single night in Pursat, Preah Vihear, and Ratanakiri provinces in Cambodia. *Horizontal line* and *error bars* indicate mean and SD. The trapping methods included: *CBT* cow-baited tent, *HBT* human-baited tent, *HLC* human landing collection, F-I (fence facing village), F-O (fence facing away from village), F (both sides of fence combined—Ratanakiri only), LT (CDC light trap), LT-M (CDC light trap baited with molasses and yeast). The *asterisks* represent comparisons of trap effects on total anopheline catch by ANOVA and Tukey’s multiple comparison test where *p ≤ 0.05, **p ≤ 0.01, ***p ≤ 0.001, and ****p ≤ 0.0001. *NB*: this figure includes all anophelines collected, not all of which were molecularly analysed for species identification and *Plasmodium* infection

Twenty *Anopheles* species were collected over six nights in Angkrong Village, Pursat (Table 1), where trap method significantly affected capture rates (F = 33.6; df = 6, 30; p < 0.0001, ANOVA) (Fig. 2). The CBT trapped significantly more anophelines per night than the other six methods (p ≤ 0.0001, Tukey’s HSD test). The HLC captured significantly more anophelines per night than the F-I and F-O (p ≤ 0.05), and LT and LT-M (p ≤ 0.01).

Nineteen *Anopheles* species were collected over six nights in Chean Mok Pagoda Village, Preah Vihear (Table 1), where trap method also significantly affected capture rates (F = 61.6; df = 6, 30; p < 0.0001) (Fig. 2). The CBT trapped significantly more anophelines per night than all other methods (p ≤ 0.0001). The HBT and HLC captured similar numbers of anophelines per night, and showed a non-significant trend in trapping about four-fold more anophelines per night than the F-I, F-O, LT, and LT-M.

An extremely diverse set of 25 *Anopheles* species was collected over only four nights (due to extreme weather conditions) in Sayas Village, Ratanakiri (Table 1), where trap method also significantly affected capture rates (F = 19.71; df = 5, 15; p ≤ 0.0001) (Fig. 2). The CBT trapped significantly more anophelines per night than all other methods (p ≤ 0.0001). The HBT and HLC captured similar numbers of anophelines per night, and showed a non-significant trend in trapping about fivefold more anophelines per night than the F-I, F-O, LT, and LT-M.

The species distributions between the three sites were relatively consistent (Tables 1, 2), but there were some exceptions. For example, only a single representative of the *An. hyrcanus* group, *Anopheles peditaeniatus*, was collected in Preah Vihear, while 3 and 5 other members of this group were collected in Pursat and Ratanakiri, respectively. Also, while distinct members of the *An. funestus* group were collected in Pursat and Preah Vihear, all of them were represented in Ratanakiri. Ongoing longitudinal collections at these sites will likely resolve these apparent differences in species distribution with more sampling points over time (Table 1).

Of the 206, 664, and 439 individual mosquito blood meals typed from collections in Pursat, Preah Vihear, and Ratanakiri, more than 97 % were from cow, 1.5 % were from cow and another host (e.g., human, goat, or pig), and 0.8 % were from human only. Over 99 % of *Anopheles* mosquitoes collected in the CBT were blood-fed on a cow (see Additional file 1). Each of the 27 species captured, including *An. dirus*, were found to be bloodfed on a cow.

All 2297 molecularly-identified specimens (Table 1) were analysed for *Plasmodium* infection by PCR [16]. Nine (0.4 %) of these specimens representing five distinct *Anopheles* species were positive for *P. falciparum* (Table 3), and all were captured in the CBT. These positive specimens included one *An. hyrcanus* in Pursat, and one *An. barbirostris*, two *An. barbirostris* (clade III) (a newly described cryptic species [19]), one *An. hyrcanus*, three *An. nivipes*, and one *An. peditaeniatus* in Ratanakiri; no positive specimens were found in Preah Vihear. Both of the *An. hyrcanus* specimens collected in this study (one from Pursat, one from Ratanakiri) were positive for *P. falciparum*. The infection rates of the other four species were 1.4, 3.9, 4.8, and 25 % (Table 3). Further sampling and analysis of a greater number of field-collected female anophelines are needed to determine the natural infection rates of these and other species.

**Table 3:** Individual *Plasmodium falciparum*-positive *Anopheles* specimens

Molecular species	Morphological species	Positive/total	Province	Hour
*An. hyrcanus*	*An. hyrcanus gr.*	1/1	Pursat	8–9 p.m.
*An. barbirostris s.s.*	*An. barbirostris*	1/4	Ratanakiri	7–8 p.m.
*An. barbirostris clade III*	*An. barbirostris*	2/51	Ratanakiri	5–6 a.m.
*An. barbirostris clade III*	*An. barbirostris*			
*An. hyrcanus s.s.*	*An. hyrcanus*	1/1	Ratanakiri	4–5 a.m.
*An. nivipes*	*An. philippinensis*	3/219	Ratanakiri	2–3 a.m.
*An. nivipes*	*An. philippinensis*			4–5 a.m.
*An. nivipes*	*An. philippinensis*			4–5 a.m.
*An. peditaeniatus*	*An. nigerrimus*	1/21	Ratanakiri	10–11 p.m.

Molecular and morphological species identification, total positive for *Plasmodium falciparum* over the number of that species tested for *P. falciparum* infection by PCR, province of collection, and hour of collection are shown. All *P. falciparum*-positive specimens were collected in cow-baited tents

## Discussion

This study demonstrated that CBTs attract much higher numbers of anophelines than HLCs or HBTs, capturing hundreds of *Anopheles* mosquitoes in a single night without risking human exposure to potentially infectious bites, as do standard HLCs. During a short collection period in only three villages across Cambodia, CBTs captured a broad range of anopheline species that are known to be human-biting in Cambodia as well as many that are considered animal-biting or catholic in their feeding habits. In the GMS, the low HLC sampling rates observed in this and other studies do not justify the use of HLC as a regular sampling and monitoring tool. There are some indications that providing prophylaxis to collectors during and immediately following collection periods reduces malaria incidence [24], when HLC is the most effective means of collecting vectors. However, prophylaxis does not prevent other common vector-borne diseases in the GMS, such as Japanese encephalitis, dengue, and filariasis. Alternatives to HLC are needed in Southeast Asia to mitigate the risk of volunteer exposure to potentially-infectious bites and to efficiently and cost-effectively monitor malaria vectors.

Light traps have been proposed as a more cost-effective way to collect mosquitoes. In many regions, however, LTs have been found to sample a different species distribution than HLCs, while also catching far fewer mosquitoes [25, 26], and the efficacy of LT captures have been shown to vary by location [27, 28]. In this study, LTs caught very low numbers of anophelines in locations where hundreds of other anopheline mosquitoes were caught per night.

While all of the *Anopheles* species collected in each of the trapping methods evaluated were also collected in the CBT, four additional (*Anopheles pallidus*, *An. barbirostris*, *An. hyrcanus*, and *Anopheles nitidus*) and one unknown *Anopheles* species were collected only in the CBT. This finding is useful because it suggests that the CBT will enable entomologists to screen higher numbers of a broader range of *Anopheles* species for *Plasmodium* infection than with any other sampling method tested here. The high rate of cow blood-fed mosquitoes was likely due both to the high attractiveness of the cow due to a greater body size and CO_2_/odor output, and the availability of the cow for a sustained and successful blood feed. The majority of CBT-captured mosquitoes were bloodfed and 99 % of all of the bloodfed mosquitoes captured in any of the traps were bloodfed on a cow.

The presumed major vector in the GMS, *An. dirus A*, comprised <1 % of the total collection and was just as scarce in the HLC and HBT collections. While major seasonal shifts in *Anopheles* species and vector composition do occur in many localities, it is unlikely that *An. dirus* is the major vector throughout the year and across different ecotypes in the GMS. The most abundant species captured in this study, *An. nivipes*, *An. vagus*, *An. kochi*, *An. barbirostris*, and *An. saeungae,* are all considered to be outdoor-biting and generalist-feeding. Despite the fact that many secondary vectors can be infected with *P. falciparum* and *Plasmodium vivax* in laboratory and field settings [13, 29–32], these vectors are still not regularly screened for infection. Since ELISA positivity can overestimate entomological inoculation rates [33], PCR analysis of DNA extracted from the head and thorax alone was used, along with sequence data, to more accurately identify *Plasmodium*-infected anophelines. None of the six distinct *P. falciparum*-infected *Anopheles* species identified would have been screened for infection in typical surveys, as they belong to species groups that are not considered major vectors in the GMS. Given the low infection rates among many of these species, further field investigations are required to assess various species’ roles in malaria transmission in Cambodia. Since all of the *Plasmodium*-infected mosquitoes identified were collected in the CBT, this trapping method is likely to be the most informative. Given the diversity and *P. falciparum* infection of *Anopheles* species captured using the CBT, interventions utilizing cow baits could be effective for targeting outdoor and generalist-feeding vectors in the GMS to reduce transmission.

The sampling sites selected for this study represent regions with a very complex outdoor transmission system, where more than 20 *Anopheles* species were present at a single site, and where bed nets and indoor residual sprays may not target GMS vectors that are active and host-seeking when people are outdoors [34]. This sampling effort represents only a single time point in each province during the high malaria transmission season. Efforts to comprehensively evaluate the seasonal and spatial distribution of malaria vector species over an entire year in Cambodia are underway.

Malaria elimination in the GMS will require sampling methods that screen all potential vector species, particularly those vectors that bite and rest outdoors where transmission is occurring. The effective and unbiased evaluation of diverse vectors in this region will be critical for the containment of multidrug-resistant parasites emerging and spreading from Cambodia.

## Conclusions

This study shows that cow-baited tents were able to capture high numbers of similar and diverse *Anopheles* species when compared to human landing collections and other commonly used collection methods, in three distinct Cambodian provinces. In the Greater Mekong Subregion, where malaria transmission occurs outdoors, this strategy may provide an effective alternative to human landing collections for sampling diverse outdoor malaria vectors.

## Additional file

10.1186/s12936-016-1488-y Bloodmeal analysis of bloodfed anophelines, according to trap type, province, and species. The data provided represent identification of animal sources of blood meals found in individual *Anopheles* mosquitoes. These were identified using a multiplex PCR assay, and are grouped according to each mosquito’s province of collection and molecular species identification.

## Abbreviations

GMS: Greater Mekong Subregion
CBT: cow-baited tent
HBT: human-baited tent
HLC: human landing collection
F: barrier fence collection
F-I: village (inner)-facing side of the barrier fence
F-O: forest (outer)-facing side of the barrier fence
LT: CDC-miniature light trap
LT-M: CDC-miniature light trap baited with yeast and molasses
