# Supplementary material for: Cow-baited tents are highly effective in sampling diverse Anopheles malaria vectors in Cambodia
Source: Malar J. 2016 Aug 30;15(1):440. doi: 10.1186/s12936-016-1488-y (PMC5004278; doi:10.1186/s12936-016-1488-y)
Supplement: Supplementary file 1 — 10.1186/s12936-016-1488-y Bloodmeal analysis of bloodfed anophelines, according to trap type, province, and species. The data provided represent identification of animal sources of blood meals found in individual Anopheles mosquitoes. These were identified using a multiplex PCR assay, and are grouped according to each mosquito’s province of collection and molecular species identification. [file 12936_2016_1488_MOESM1_ESM.docx]

**Supplementary Table 1.** Bloodmeal analysis of bloodfed anophelines, according to trap type, province, and species.


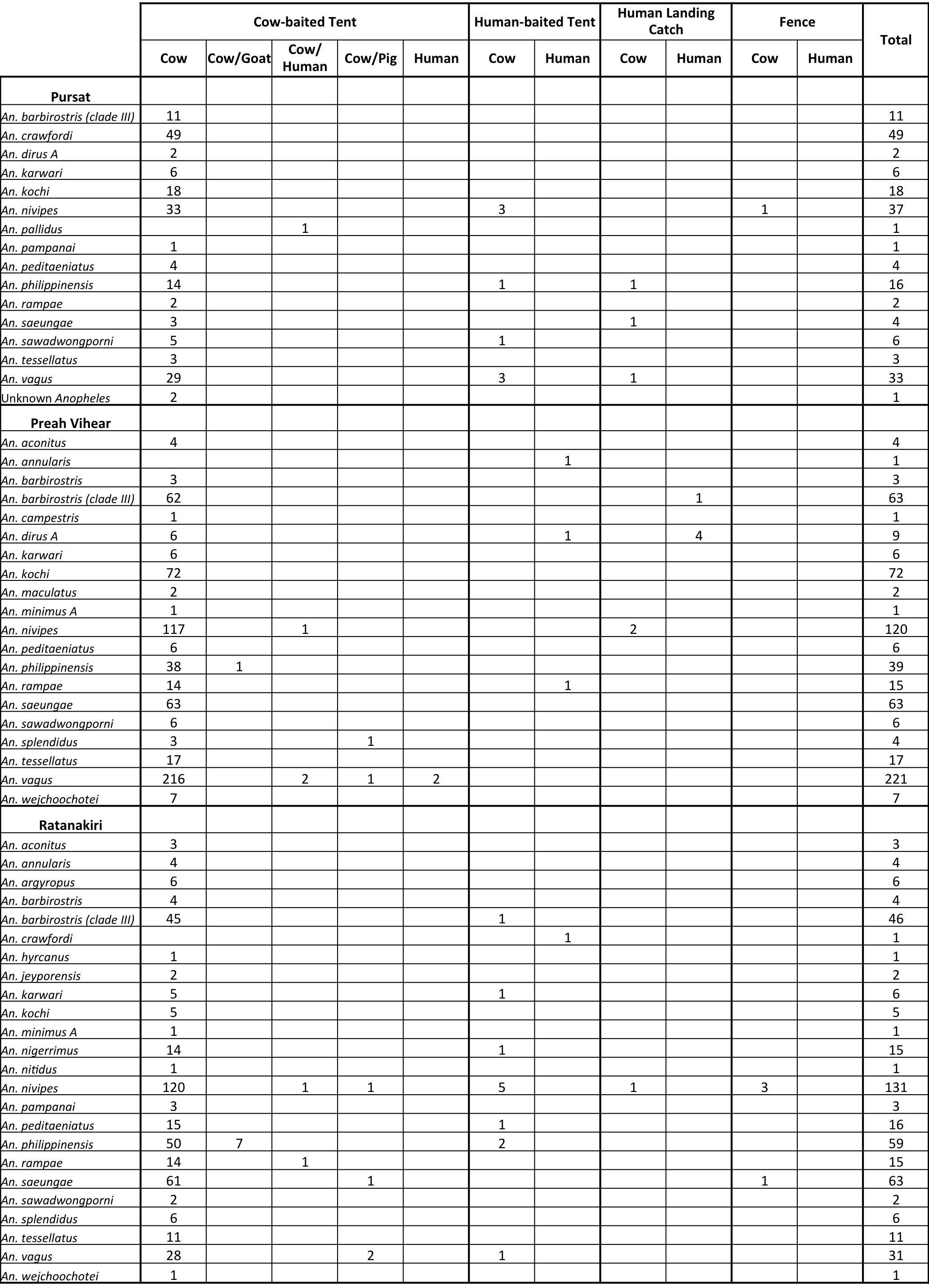


**Supplementary Table 1. Bloodmeal identification of anophelines captured in cow-baited tents, human-baited tents, human landing collections, and barrier fences.** The animal sources of blood meals were identified using a multiplex PCR assay, and are grouped according to each mosquito’s province of collection and molecular species identification. No bloodfed specimens were collected in the CDC light trap collections, and blood meal types and bloodmeal combinations that were not found are not included as columns in the table. The total number of bloodfed specimens for each species is shown in the last column.
